# Clinically unsuspected pyelonephritis in children younger than 7 years

**DOI:** 10.1007/s10140-025-02375-w

**Published:** 2025-08-16

**Authors:** Boaz Karmazyn, David S. Hains, Rebeca Santos, S. Gregory Jennings, George J. Eckert, Rosalia Misseri

**Affiliations:** 1 Department of Radiology and Imaging Sciences, Riley Hospital for Children, Riley Hospital for Children at Indiana University Health, Indiana University School of Medicine, 705 Riley Hospital Dr., Rm 1053, Indianapolis, IN 46202 USA; 2 Department of Pediatric nephrology, Riley Hospital for Children, Riley Hospital for Children at Indiana University Health, 705 Riley Hospital Drive, Room 230, Indianapolis, IN 46202 USA; 3 Department of Indiana University School of Medicine, 340 W 10th St, Indianapolis, IN 46202 USA; 4 Department of Radiology and Imaging Sciences, Indiana University, 950 W. Walnut St. Rm E124, Indianapolis, IN 46202 USA; 5 Department of Biostatistics and Health Data Sciences, Indiana University School of Medicine, 340 West 10th Street Fairbanks Hall, Suite 6200, Indianapolis, IN 46202 USA; 6 Department of Pediatric Urology, Riley Hospital for Children at Indiana University Health, Indiana University School of Medicine, 705 Riley Hospital Drive, Room 4230, Indianapolis, 46202 IN USA

**Keywords:** Pyelonephritis, Abdomen CT, Urinalysis, Urine culture

## Abstract

**Purpose:**

Diagnosis of pyelonephritis can be challenging in young children. Our purpose is to evaluate the incidence and characteristics of CT-diagnosed pyelonephritis that was not clinically suspected in children under 7 years of age.

**Methods:**

We retrospectively identified children < 7 years with CT diagnosis of pyelonephritis between 2011 and 2024. Demographic, clinical, and laboratory data were extracted from the medical record. One pediatric radiologist reviewed all CT scans and recorded the findings. Wilcoxon rank sum tests were used to compare age with clinically unsuspected pyelonephritis and negative urinalysis; Chi-square tests compared extent of pyelonephritis with renal atrophy and dilated (grades 3–5) VUR.

**Results:**

104 children (mean age 4.8 years; 79 females) met inclusion. 92/104 (88.5%) had no UTI history; 34/104 (32.7%) had urinary symptoms. Pyelonephritis was clinically unsuspected in 53/104 (51.0%), with no age group difference (*p* = 0.579). Urinalysis was negative in 17/104 (16.3%). 26 children received antibiotics prior to sampling. CT showed pyelonephritis in 126 kidneys (48 right, 34 left, 22 bilateral); 7 children had renal abscesses. Renal scarring developed in 11/47 with follow up renal imaging (23.4%). VUR was found in 41/51 with voiding cystourethrogram (80.4%), including 26 with grade 3–5 VUR. No association was found between extent of renal involvement and atrophy/scarring (*p* = 0.978) or VUR (*p* = 0.751).

**Conclusion:**

CT-diagnosed pyelonephritis in young children is often clinically unsuspected and may present with negative urine tests. Follow-up US and voiding cystourethrogram are warranted to assess for scarring and VUR, even in the absence of prior UTI.

**Graphical Abstract:**

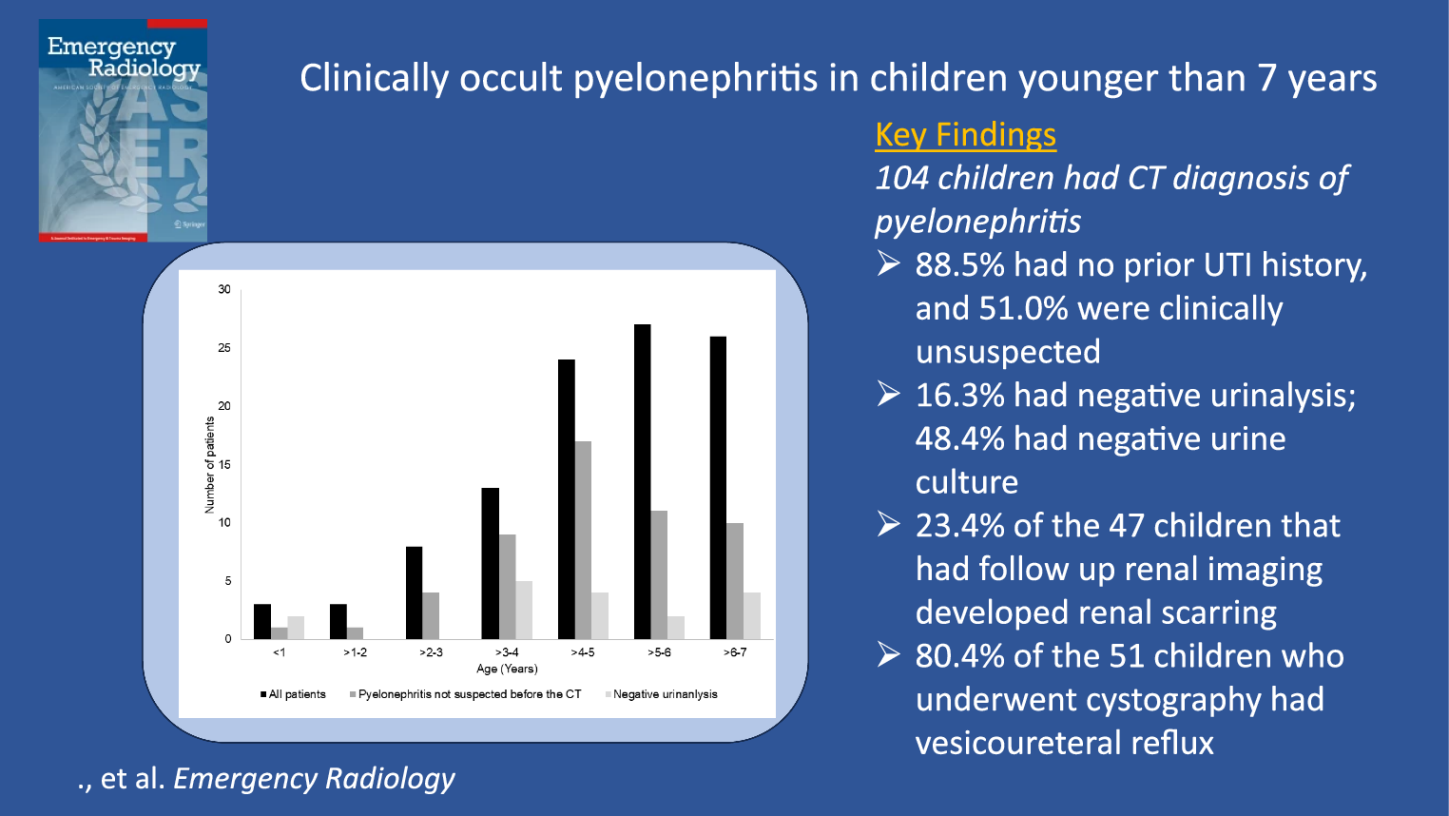

## Introduction

Acute pyelonephritis is the most common serious bacterial infection in children [1] and is a common cause for admission of children with fever of unknown origin to the ED [2]. Most children with urinary tract infection (UTI) and fever have pyelonephritis [1]. The diagnosis in most children is typically based upon clinical evaluation, positive urinalysis, and urine cultures [3]. But diagnosing young children can be challenging due to limited verbal communication and non-specific symptoms such as fever, abdominal pain, and vomiting.

CT is a sensitive imaging technique for evaluation of acute abdominal pathologies, most commonly appendicitis. CT is typically performed after an equivocal or negative US in children with acute abdominal pain [4]. CT is sensitive for evaluation of renal pathologies, and an animal study demonstrated that CT is equally sensitive and reliable for the detection of acute pyelonephritis compared with 99mTc-DMSA [5]. A study in 36 adult patients with a clinical diagnosis of pyelonephritis suggested higher accuracy of CT as compared with 99mTc-DMSA [6]. In children with a clinical diagnosis of pyelonephritis, CT has the advantage of evaluating for a renal abscess and alternative diagnoses. The findings of pyelonephritis in CT include striated enhancement, hypodensity, perirenal stranding, and uroepithelial enhancement [7, 8].

There are only a few studies of children diagnosed with pyelonephritis based on CT [8, 9]. Given that the clinical diagnosis of pyelonephritis in young children can be challenging, we wanted to evaluate the incidence and characteristics of CT-diagnosed pyelonephritis that was not clinically suspected in children under 7 years of age.

## Materials and methods

### Patient population

This was a HIPAA-compliant retrospective (1/2011-12/2024) single-center study performed at a tertiary children medical center and approved by the institutional review board with waiver of written informed consent. We searched the institutional radiology information system (RIS) for all children younger than 7 years-old admitted to the emergency department or transferred for admission to the hospital with acute symptoms, and who underwent abdominal CT with a diagnosis of pyelonephritis in the radiology report. The electronic medical records (EMR) were reviewed for demographic information, underlying medical conditions, history of prior UTI, medications prior to admission, clinical presentation, and physical examination findings of the abdomen and flanks.

The indication for the abdominal CT was based on the CT order and EMR records. We defined children in whom pyelonephritis was not suspected if the CT order or EMR prior to the CT did not include the possibility of UTI or pyelonephritis in the diagnostic considerations.

We excluded children with prior urologic surgery, children with neurogenic bladder, chromosomal abnormalities, severe developmental delay, and history of abdominal trauma in the last four weeks.

Using the RIS, we also identified all children < 7 years-old evaluated in the ED for abdominal pain, fever, or appendicitis during the study period.

### Laboratory results

Leukocytosis was defined as white blood cells > 13,500 cell/µL. Positive urinalysis was considered as any of the following: any number of bacteria, positive leukocyte esterase, white blood cells per high-power field > 5, or positive nitrite [3].

Urine culture was considered positive if > 50,000 CFUs per mL [3].

### CT of the abdomen technique

Abdominal CT was performed with intravenous contrast Iopamidol (Isovue-3104, Bracco Diagnostics) 1 mL per kg body weight. CT scans were performed using automated current selection using either Brilliance iCT256 (Philips Medical Systems, Cleveland, OH] with a kVp of 80 or 100, or SOMATOM Force (Siemens Healthcare, Forchheim, Germany) with automated kilovoltage selection (Siemens CARE KV, Siemens Healthcare). All CT scans were reconstructed in the axial plane (slice thickness of 4 and increment of 3 mm with soft tissue and lung kernels), and multiplanar reconstructions in the coronal (slice thickness of 4 mm, 3 mm increment) and sagittal (slice thickness and increment of 3 mm) planes.

### Review of the abdominal CT

The abdominal CT scans were reviewed by a fellowship-trained pediatric radiologist with 25 years of experience. Each kidney and ureter were evaluated for the following abnormalities:

- Atrophy - length less than two standard deviations expected by the child’s age.
- Scarring - focal thinning of the cortex.
- Swelling - enlarged contour of abnormal enhancing parenchyma, either focally or diffuse.
- Striated nephogram - alternating linear bands of high and low attenuation in a radial pattern extending through the corticomedullary layers of the kidney.
- Hypodensity - decreased attenuation with no striated nephrogram.
- Uroepithelial enhancement - enhancement of the pelvic or ureteral urothelium.
- Perirenal fat stranding - increased density of the perirenal or pararenal fat.
- Abscess – well-defined fluid collection in the renal parenchyma.

The number of pyelonephritic foci in each kidney was recorded. More than four locations were considered multifocal, and when the entire kidney was involved, defined as diffuse pyelonephritis.

### Statistical evaluation

Descriptive statistics were used to evaluate demographic characteristics of the patient population, clinical presentation, laboratory results, and imaging findings. Wilcoxon rank sum tests were used to compare ages in children with negative laboratory results for UTI in children with occult clinical diagnosis of pyelonephritis. Chi-square test was used to compare the extent of pyelonephritis (1, 2, 3, 4, > 4 focuses and diffuse involvement) of the kidneys versus more extensive involvement, and the outcomes of renal atrophy and dilated vesicoureteral reflux (VUR, grades 3–5). In cases of bilateral pyelonephritis, only the kidney with the more extensive involvement was included in the analysis. A two-sided 5% significance level was used for all tests. Statistical analyses were performed using SAS (version 9.4, SAS Institute).

## Results

### Patient population

**Fig. 1:**
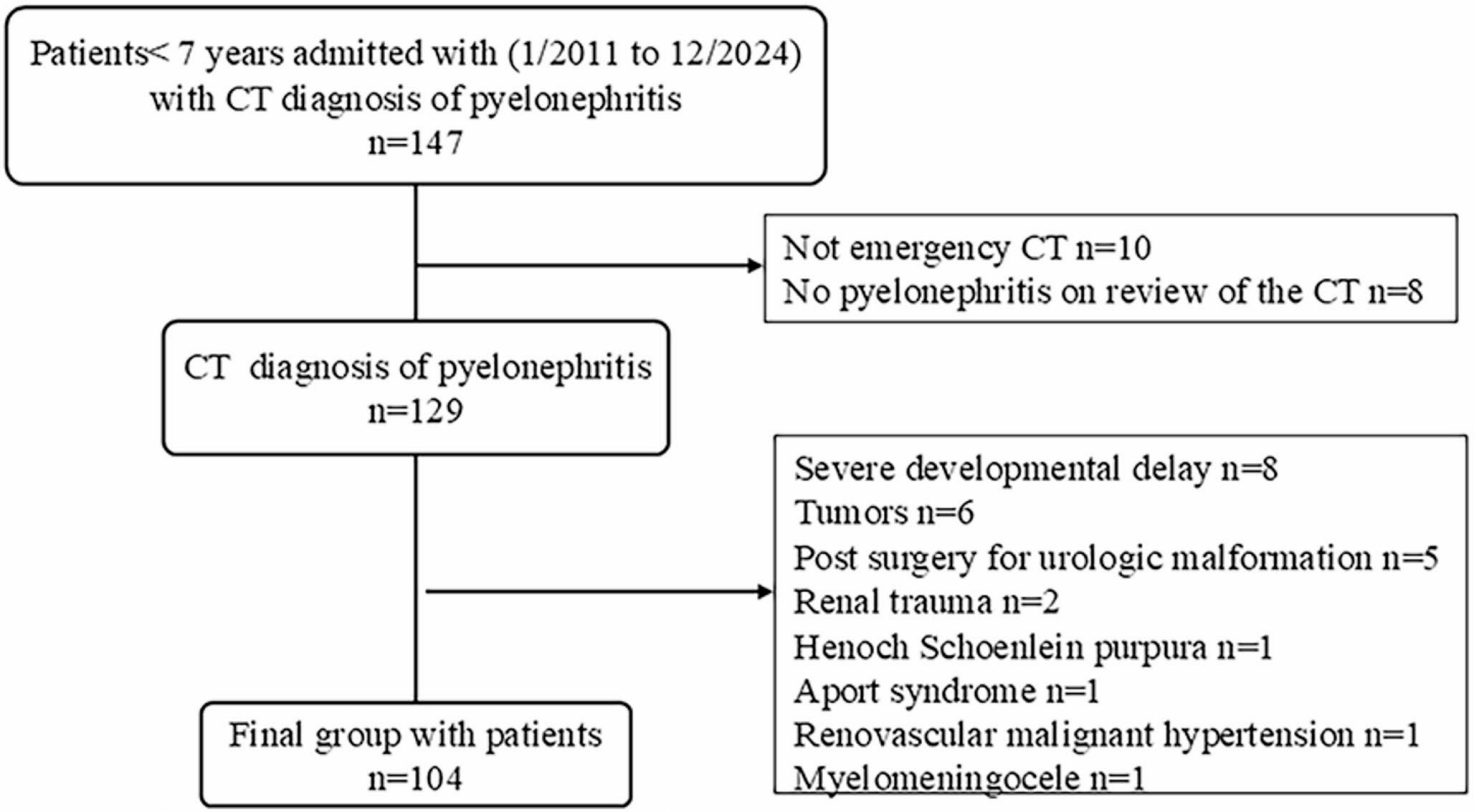
Flow chart of children accepted to the study

From January 2011 to December 2024, 147 children had a diagnosis of pyelonephritis noted in the CT report. 43 children were excluded for the following reasons. In 10 children CT was not performed in the emergency department, and they were not admitted to the hospital for acute symptoms related to pyelonephritis. On retrospective review of the CT there was no evidence of pyelonephritis in eight children, three of whom had renal scarring and none of them had clinical diagnosis of UTI or pyelonephritis. Eight children had severe developmental delay due to cerebral palsy (*n* = 4) or chromosomal abnormalities (*n* = 4). Six children had tumors and were treated with chemotherapy. Five children had prior surgery for urologic anomalies. Two children had a history of recent trauma. One child had Henoch Schoenlein purpura. One child had Aport syndrome. One child who presented with malignant hypertension had acute renal ischemic changes secondary to renal artery stenosis due to fibromuscular dysplasia. One child had history of a myelomeningocele with neurogenic bladder (Fig. 1).

The final study group included 104 children (79 females and 25 males) younger than seven years with an average age of 4.8 years.

19 children had a history of prematurity (gestational age range from 30 to 36 weeks). 12 children had a history of UTI, and two had VUR.

### Clinical presentation and physical examination findings

The most common presenting symptoms were fever (91/104, 87.5%), abdominal pain (82/104, 78.8%), and vomiting (59/104, 56.7%). Only five of the 104 children had flank or back pain (4.8%) and 34 children (32.7%) had one or more of the following urinary symptoms: dysuria (*n* = 17, 16.3%), urinary frequency (*n* = 6, 5.8%), urinary incontinence (*n* = 5, 4.8%), decreased urine output (*n* = 3, 2.9%), odorous urine (*n* = 1) and penile pain (*n* = 1). Ten of the 104 children (9.6%) had diarrhea, three (2.9%) had cough, three (2.9%) had headaches, and three (2.9%) had hip pain.

On physical examination, the most common findings were right lower quadrant tenderness (47/104, 45.2%) and diffuse abdominal tenderness (13/104,12.5%). Flank, back, or suprapubic tenderness was found in 21 of the 104 children (20.2%); flank or back tenderness were found in 13 (12.5%) and nine (8.7%) had suprapubic tenderness.

In 33/104 children (31.7%), US was performed to evaluate for appendicitis before the CT scan.

In 34/104 (32.7%) the primary indication for the CT was to evaluate for pyelonephritis. In 17/104 (16.3%) children the diagnosis of appendicitis and either UTI or pyelonephritis were considered before the CT scan.

**Fig. 2:**
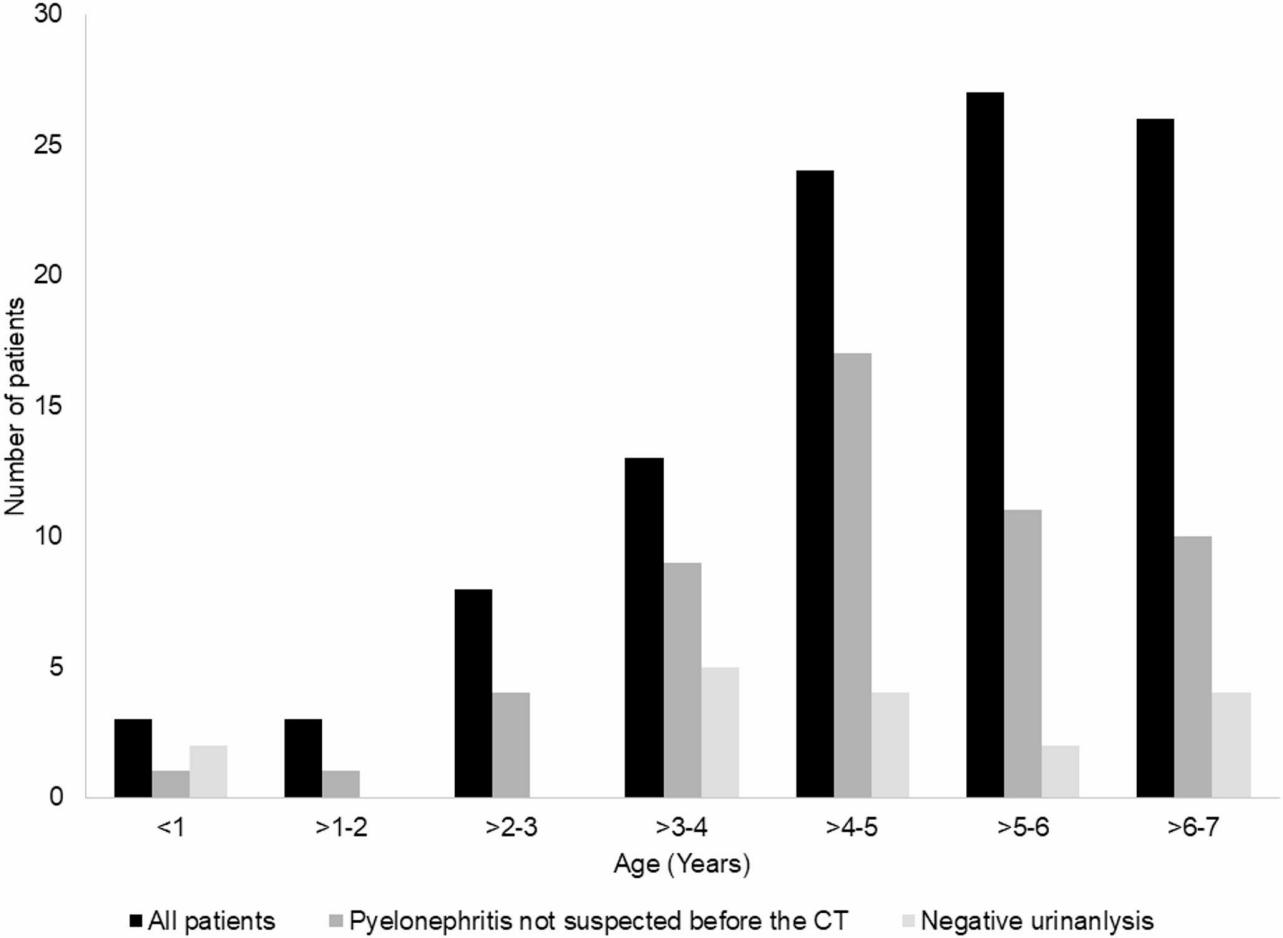
Cases of pyelonephritis not suspected clinically and with negative urinalysis in the different age groups

In 53/104 children (51.0%), a diagnosis of UTI or pyelonephritis was not in the differential diagnosis before the CT scan (Figs. 1 and 2). In 38 of these 49 (77.6%), appendicitis was the primary indication. In the other 14 children, the indication for CT scan was related to symptoms and no specific diagnosis was indicated in the medical records: generalized abdominal pain with fever (*n* = 8), right lower quadrant pian (*n* = 3), fever and vomiting (*n* = 1), fever and rectal pain (*n* = 1), and bloody stool (*n* = 1). In one child CT was performed to evaluate for a false diagnosis of pelvic cystic mass by US. Most children (73/104, 70.2%) were admitted for treatment of pyelonephritis ranging from 1 to 13 days (average 3.5 days).

### Laboratory results

Most (81/104, 77.9%) of the children had leukocytosis. Urinalysis was negative in 17/104 children (16.3%). Urine culture was obtained in 96 of the 104 children (92.3%) and in 46 (48.4%) of the children, urine culture was either negative (*n* = 34), showed colony counts < 50,000 CFU/mL (*n* = 4), or was contaminated (*n* = 8) and positive with colony counts > 50,000 CFU/mL in 43 (51.6%). *Escherichia coli* was the most common pathogen (36/49, 87.8%). Other pathogens included *Enterococcus sp.* (*n* = 4), *Proteus mirabilis* (*n* = 1) and *Klebsiella sp.* (*n* = 1), *Staphylococcus aureus* (*n* = 1).

In four of the 49 children with positive cultures, the urinalysis was normal. Thus, per American Academy of Pediatrics (AAP) criteria, in only 45/104 patients (43.3%) did the lab results confirm the diagnosis of UTI. In four other children (4/104, 3.8%), the culture was less than 50,000 CFUs per mL and in eight children (8/104, 7.7%) the urine was contaminated.

Negative urinalysis (*p* = 0.182) and negative urine culture (*p* = 0.638) were not significantly associated with age.

26 children received antibiotics before the urinalysis and urine culture. Urinalysis was positive in 21 children and urine culture was positive in 14 children. Only 12/26 (46.2%) had both positive urinalysis and urine culture.

### CT findings

**Fig. 3:**
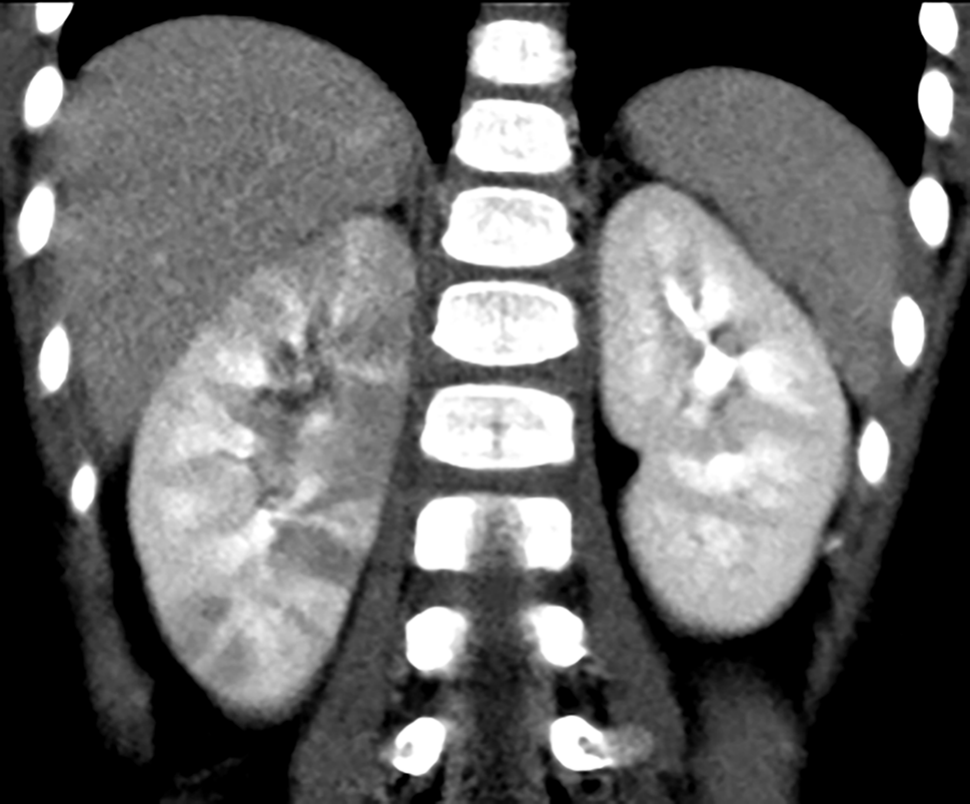
A 3 year-old female presented with fever, cough, vomiting, diarrhea, and leukocytosis (14,900 WBC/mL). Urinalysis and culture obtained from bladder catheterization were negative. A CT abdomen was obtained, due to concern for appendicitis, that demonstrated right-sided pyelonephritis. Coronal post-contrast CT demonstrates multifocal wedge-shaped hypodensities with striated nephrogram, and diffuse swelling of the right kidney

**Fig. 4:**
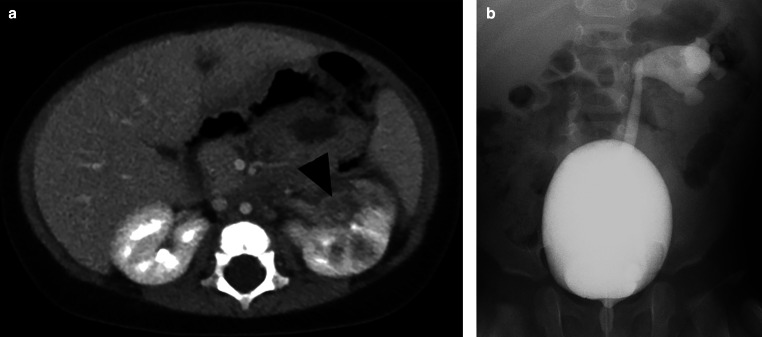
A 6 month-old boy who presented with fever, intermittent fussiness, and leukocytosis (39,000 WBC/mL) was initially evaluated with US for intussusception. Urinalysis was negative on admission and later urine culture was positive for *E. coli.* CT of the abdomen was obtained to evaluate for a mass or inflammatory process; a diagnosis of pyelonephritis was not considered. Axial post-contrast CT (**A**) shows diffuse swelling and multifocal hypodensities in the left mid-pole kidney, and a round hypodensity in the anterior cortex with wall enhancement compatible with an abscess (arrowhead). A voiding cystourethrogram (**B**) demonstrates left grade 5 vesicoureteral reflux with a droopy lily sign compatible with a reflux to a lower pole of a duplex kidney. Due to recurrence of UTIs, he subsequently had circumcision and was placed on daily antibiotics. A left ureteral reimplantation was performed after he developed recurrent pyelonephritis

Pyelonephritis was found in 126 kidneys: right (*n* = 48), left (*n* = 34), and bilateral (*n* = 22). The most common findings were hypodensity (*n* = 109), striated nephogram (*n* = 89), urothelial enhancement (*n* = 50), perirenal stranding (*n* = 46), and renal abscesses (*n* = 7, Figs. 3 and 4). Other findings included hydronephrosis (*n* = 13) and renal scarring (*n* = 12). Only three children with renal scarring had a prior history of UTIs. One child had a solitary kidney. In one child, in addition to pyelonephritis, tip appendicitis was diagnosed and confirmed by surgery.

There was no significant association (*p* = 0.579) between age and clinical occult pyelonephritis.

### Follow-up

Clinical follow-up records of at least four weeks were available in 77 of 104 children (74.0%) with an average of 2.9 years (range 28 days to 10.9 years). 12/104 children (11.5%) with no history before the admission of prior UTI had recurrent UTI, 16 (15.4%) had functional bladder abnormality, and 5 (4.8%) had recurrent pyelonephritis.

Follow-up renal imaging was performed at least four weeks after the initial CT scan in 47 of 104 children (45.2%). Of these, 30 underwent only renal ultrasound, 10 had ultrasound plus either follow-up CT (*n* = 4), DMSA (*n* = 5), or both (*n* = 1), and 7 underwent only follow-up CT. Ultrasound (*n* = 40) revealed new renal scarring in five kidneys. Additional imaging confirmed scarring in two patients (one by DMSA and one by both DMSA and CT) and detected new scarring in four others (one by DMSA and three by CT). Among the seven children who had only CT follow-up, scarring was identified in two additional patients. Overall, 11 of 47 children (23.4%) had new renal scarring on follow-up imaging.

Seven follow up CT scans on other patients demonstrate renal scarring in 2 additional patients. Overall, 11 of the 47 children (23.4%) had new renal scarring on follow-up imaging studies.

VCUG was performed in 51 of the 104 children (49.0%), of whom 41 had VUR (80.4%); 26 of them had dilated (grade 3 to 5) VUR (63.4%). In addition, one child had bilateral Hutch diverticula, and another child had a small right periureteral diverticula. Six female children had spinning top urethra indicating functional bladder abnormality, and one child had trabeculated bladder secondary to bladder functional abnormality. Two children had duplex kidneys with reflux to the lower moieties (Fig. 2).

There was no significant difference between degree of renal involvement and renal scarring (*p* = 0.978) or dilated VUR (*p* = 0.751).

18 children were treated with either unilateral (*n* = 7) or bilateral (*n* = 11) ureteral reimplantation, and four children were treated with bilateral Deflux injection. One child had an incision of a small posterior urethral valve.

## Discussion

Our study shows that in nearly half of children younger than seven years (46.2%), the diagnosis of pyelonephritis was not considered before the abdominal CT scan, and the most frequent indication to perform a CT scan was to evaluate for appendicitis. Whether pyelonephritis was considered was not statistically correlated with the child’s age. A few possible explanations exist for not considering pyelonephritis prior to the CT scan in these children. In most of our children, the symptoms were not localized to the urinary tract. Only about a third (32.7%) of the children had urinary symptoms. In addition, the diagnosis of pyelonephritis is typically based on clinical and laboratory evaluation, and in only a few children the diagnosis is based on the CT scan. As far as we know there is only one prior study reporting CT diagnosis of pyelonephritis not suspected clinically, in 12 children; two (16.7%) had negative urine culture [9].

In 16.3% of the children, urinalysis was negative. Our results of false-negative urinalysis and urine cultures are in line with prior studies. A meta-analysis of 95 studies in 95,703 children demonstrated that negative urinalysis is found in around 10% of children with UTI, with increased sensitivity with the use of microscopy to detect pyuria and the presence of bacteria [10].

In 48.4% of the children, urine culture was either negative, showed colony counts < 50,000 CFU/mL, or was contaminated. One possible explanation for the negative laboratory studies is prior antibiotic treatment. In our study 26 children had documentation of treatment with antibiotics prior to the laboratory workup, and only 14 of them had positive urine cultures. It is possible that the use of antibiotics in children prior to ED presentation was underreported and contributed to some of the false-negative laboratory studies [11]. False-negative urine cultures have been documented in other studies. A study in women showed that depending on the method of obtaining urine culture, urine cultures can be positive in only 33–84% of patients with UTI [12]. In a study of newborns, 24.7% had false-negative urine culture [13]. To avoid overtreatment of asymptomatic bacteriuria, AAP criteria for confirmation of UTI require both positive culture of at least 50,000 CFUs/mL, and pyuria and/or bacteriuria [3]. In our study, 43.3% of the children with CT diagnosis of pyelonephritis met the AAP criteria for UTI. Negative urinalysis was correlated with younger age. Negative culture was not correlated with child age.

The most common findings in the CT study included focal areas of hypodensity, striated nephogram, uroepithelial enhancement, and perirenal fatty standing. Seven of the children had renal abscesses, which is comparable to findings from other studies [6, 7, 14]. In 12 children there was renal scarring indicating prior episodes of pyelonephritis, three of them had a prior history of UTI.

Most children in our study (70/2%) were admitted to the hospital. This is comparable to the data from a nationwide sample on 1,904,379 children presenting to the ED with UTI. It demonstrated that the admission rate was higher in younger children, with a 57.9% admission rate in children younger than one year [2].

On follow-up, 11.5% of children with no documented UTI before the CT was performed had recurrent UTIs, and 20.8% were found to have a functional bladder abnormality.

New renal scarring and atrophy developed in 11 of the 47 children (23.4%) who underwent follow-up renal imaging. However, as 30 of these children had only ultrasound—an imaging modality with lower sensitivity compared to CT or DMSA—this likely represents an underestimation of the true risk of renal scarring [14].

VUR was detected in 80.4% of the children that had VCUG, with most of these having dilated VUR (63.4%). This is a higher incidence compared with the reported VUR in children with UTI [3, 15]. This is likely an underestimation of the true risk of VUR as only about half of the patients had a VCUG study.

There was no correlation between the extent (one focus versus more extensive involvement) of pyelonephritis in CT and follow-up findings of renal atrophy or dilated VUR.

Our study has several limitations related to retrospective analysis. It is possible that not all of the pertinent clinical information, including prior UTIs and recent treatment with antibiotics, were obtained in the ED or on admission. In most children the urine specimen was obtained through midstream clean catch method which is associated with increased urine contamination. However, in our study only 7.7% of the urine specimens were contaminated, which is less than reported in other studies [16]. There was no pathologic confirmation or any other gold standard evaluation of CT diagnosis of pyelonephritis, and the authors assumed the high accuracy of CT diagnosis in detection of pyelonephritis as documented in prior studies [5, 6, 17]. Most children did not have follow-up US, and as only about half of the children had a VCUG study, the number of children that were complicated with renal atrophy and had VUR may be an underestimationIn addition, the use of CT to evaluate pyelonephritis introduces a selection bias toward more severe cases and therefore these findings cannot be generalized to all children diagnosed clinically with pyelonephritis.

In conclusion, pyelonephritis was commonly (46.2%) not considered prior to abdominal CT in children with acute pyelonephritis. Appendicitis was the most common mimicker of pyelonephritis in children age < 7 years who are diagnosed by CT. Our preliminary study suggest that children with CT diagnosis of pyelonephritis are at risk of renal scarring and VUR regardless of urinalysis findings or history of UTI and should be followed with US and voiding cystourethrogram. It is concerning that most children did not have follow-up US and only half of them had a VCUG study.

## Data Availability

Anonymized data that support the findings of this study are available on request from the corresponding author. The data are not publicly available due to privacy or ethical restrictions.
